# The prevalence of clinically-relevant comorbid conditions in patients with physician-diagnosed COPD: a cross-sectional study using data from NHANES 1999–2008

**DOI:** 10.1186/1471-2466-12-26

**Published:** 2012-06-13

**Authors:** Kerry Schnell, Carlos O Weiss, Todd Lee, Jerry A Krishnan, Bruce Leff, Jennifer L Wolff, Cynthia Boyd

**Affiliations:** 1 Johns Hopkins University, 3400 North Charles Street, Baltimore, MD, 21218, USA; 2 Hines VA Hospital & University of Illinois at Chicago, 5000 South 5th Ave, P.O. Box 5000, Hines, IL, 60141, USA; 3 University of Illinois Hospital & Health Sciences System, Medical Center Administration Building, 914 South Wood Street, MC 973, Chicago, IL, 60612, USA

## Abstract

**Background:**

Treatment of chronic diseases such as chronic obstructive pulmonary disease (COPD) is complicated by the presence of comorbidities. The objective of this analysis was to estimate the prevalence of comorbidity in COPD using nationally-representative data.

**Methods:**

This study draws from a multi-year analytic sample of 14,828 subjects aged 45+, including 995 with COPD, from the National Health and Nutrition Examination Survey (NHANES), 1999–2008. COPD was defined by self-reported physician diagnosis of chronic bronchitis or emphysema; patients who reported a diagnosis of asthma were excluded. Using population weights, we estimated the age-and-gender-stratified prevalence of 22 comorbid conditions that may influence COPD and its treatment.

**Results:**

Subjects 45+ with physician-diagnosed COPD were more likely than subjects without physician-diagnosed COPD to have coexisting arthritis (54.6% vs. 36.9%), depression (20.6% vs. 12.5%), osteoporosis (16.9% vs. 8.5%), cancer (16.5% vs. 9.9%), coronary heart disease (12.7% vs. 6.1%), congestive heart failure (12.1% vs. 3.9%), and stroke (8.9% vs. 4.6%). Subjects with COPD were also more likely to report mobility difficulty (55.6% vs. 32.5%), use of >4 prescription medications (51.8% vs. 32.1), dizziness/balance problems (41.1% vs. 23.8%), urinary incontinence (34.9% vs. 27.3%), memory problems (18.5% vs. 8.8%), low glomerular filtration rate (16.2% vs. 10.5%), and visual impairment (14.0% vs. 9.6%). All reported comparisons have p < 0.05.

**Conclusions:**

Our study indicates that COPD management may need to take into account a complex spectrum of comorbidities. This work identifies which conditions are most common in a nationally-representative set of COPD patients (physician-diagnosed), a necessary step for setting research priorities and developing clinical practice guidelines that address COPD within the context of comorbidity.

## Background

Chronic Obstructive Pulmonary Disease (COPD) is the 4^th^ most common cause of death in the United States, with projections that it will move into 3^rd^ place by 2020. Currently, COPD is the attributable cause of death for more than 120,000 deaths per year. While deaths from stroke and heart disease decreased between 1970 and 2002, death rates for COPD nearly doubled [1]. COPD is also a leading cause of hospitalizations in older adults [2], as well as of other morbidity.

COPD does not simply contribute to mortality. It may contribute substantially to difficulties with activities of daily living and disrupt social functioning [3]. A study in 2003, for example, found the presence of either moderate or severe COPD to be associated with a higher odds ratio of functional limitations [4].

The majority of patients with COPD have more than just COPD - comorbidities in COPD are the rule, rather than the exception. A study of 200 COPD patients from a managed care organization, for example, found that 94% of patients had at least one other chronic medical condition [5]. This is significant because comorbidities in COPD are associated with poorer outcomes, both for COPD and the other conditions [6,7]. Previous studies have shown an association between a variety of chronic conditions and COPD, including hypertension, diabetes, heart failure, coronary artery disease, and malignancy [6-9].

Previous studies on comorbidities in COPD have typically focused on selected chronic medical conditions, such as heart failure and diabetes. These studies have largely failed to look comprehensively at many other high-priority conditions, such as arthritis and obesity, and important functional limitations, like cognitive impairment and limited mobility. Functional limitations can have a significant impact on the treatment of chronic conditions, as patients may have difficulty adhering to treatment regimens [10]. These conditions may also modify the effectiveness of COPD therapy, cause potentially dangerous therapeutic interactions, and make COPD therapies less feasible.

Despite these potential interactions and the complexities of clinical decision-making for people with COPD, little population–based data on the prevalence of comorbidities in COPD is available. To date, there have been no nationally-representative studies of the prevalence of comorbidities in COPD. Moreover, COPD clinical practice guidelines do not provide specific recommendations for older patients with multiple comorbid diseases [11]. Thus, in this study, we aim to describe the prevalence of clinically-relevant comorbid conditions that add to the complexity of clinical decision-making or self-management of COPD in a nationally-representative population of people with physician-diagnosed COPD. We also compare these prevalence estimates to those seen in subjects without COPD, to gain a better understanding of which conditions in particular are more common in people with COPD.

## Methods

### Study population

NHANES is a nationally-representative study designed to assess the health and nutritional status of non-institutionalized civilians in the US. Collection of information occurs through home interviews and exams in mobile centers. Study details, including operations manuals, are publicly available [12]. To ensure adequate sample size in age and gender strata, we joined five survey waves (1999–2000, 2001–2002, 2003–2004, 2005–2006, and 2007–2008). This created an analytic sample of 14,828 people age 45 and older, including 995 with COPD. Using the sampling weights described below, this sample represents around 100 million people, 10 million of whom have COPD. From 1999–2008, the NHANES interview response rates ranged from 78% to 84%. Of those interviewed, 75% to 80% completed the physical exam.

### Definition of conditions

COPD and comorbid disease status were ascertained largely through NHANES questions asking “has a doctor or other health professional ever told you that you have [disease]?” Physician-diagnosed COPD was defined as a positive response to either chronic bronchitis or emphysema with a negative response to current asthma. Subjects were defined as having a history of smoking if they reported having smoked >100 cigarettes total in their life.

Coronary heart disease (CHD) was defined by an affirmative response to at least one of CHD, angina, or heart attack. For diabetes (DM), subjects were able to report prediabetes (2007–2008) or borderline diabetes (1999–2008). Among those reporting either prediabetes or borderline diabetes, individuals were counted as having DM if they took insulin or a pill for diabetes, suffered from retinopathy, and for 1999–2004, if they had a lower extremity ulcer that took more than 4 weeks to heal, or had numbness or tingling in their hands or feet due to diabetes.

Glomerular filtration rate (GFR) was calculated using the Modification of Diet in Renal Disease (MDRD) equation based on serum creatinine, age, race, and gender. Low GFR was defined as an estimated GFR < 60 mm/L [13]. Low hemoglobin was defined as <12 g/dL in women and < 13 g/dL in men [13]. Urinary incontinence was ascertained by self-report of leaking urine at least a few times a month. Polypharmacy was defined as self-reported regular use of >4 prescription medications, following a previously established cut point [13]. Prescription dietary supplements were not counted as medications. For 1999–2000, prescription analgesics used on a chronic basis were not included in the count. Prescription analgesics were included in the drug count from 2001–2008.

Hypertension (HTN) was defined as mean systolic blood pressure ≥140 mmHg on exam, mean diastolic blood pressure ≥ 90 mmHg, and/or current use of an antihypertensive [9,14]. The mean blood pressures were calculated following NHANES protocol [12]. If there was more than one reading, the first reading was excluded from the mean; otherwise, the sole reading was considered the “mean.” Hypercholesterolemia was similarly defined by a total serum cholesterol >6.21 mmol/L or current use of a hyperlipidemia drug. Depression and anxiety were defined as self-reported current use of an antidepressant or anxiolytic, respectively.

Memory problems were defined as an affirmative response or “don’t know” to the question “are you limited in any way because of difficulty remembering or because you experience periods of confusion?” Mobility difficulty was considered present if the individual reported difficulty walking 0.25 miles or up to 10 steps without equipment. Visual impairment was ascertained through self-reported extreme difficulty reading newsprint or seeing up close, or, an examined visual acuity score of <20/50 in the better eye. Hearing impairment was defined according to self-report of “a lot” of trouble hearing or use of a hearing aid.

Individuals who reported dizziness or imbalance lasting at least 2 weeks or for an unknown duration, or, difficulty with balance in the last year, were counted as having problems with dizziness or balance. This variable was only available from 1999–2004; we present the prevalence only for the population from 1999–2004. A history of cancer was defined by self-report of having been diagnosed with cancer, excluding non-melanoma and unknown skin cancers. Subjects were considered obese if their body mass index (BMI) was ≥30 kg/m^2^.

Frailty was defined according to four of the five criteria developed in the Cardiovascular Health Study [15] and Women’s Health and Aging Studies [16], modified for NHANES [17]. Subjects from survey years 1999–2006 were defined as “frail” if they had ≥3 of the four following characteristics: low BMI, weakness, exhaustion, and low physical activity. Low BMI was defined as a BMI ≤ 18.5 kg/m^2^. Weakness was defined as a response of “some difficulty,” “much difficulty,” or “unable to do” when asked how difficult they find it to lift 10 pounds. Exhaustion was defined using these same responses when asked how difficult they find it to walk from one room to another on the same level. Low physical activity was defined as reporting less activity than other people of the same age. Physical activity relative to others of the same age was not assessed in 2007–2008. Therefore, subjects from 2007–2008 were considered frail if they had a low BMI and reported both weakness and exhaustion.

### Condition groupings

We grouped the conditions into three domains – diseases, clinical factors, and health status factors. As outlined in Boyd et al., the disease domain encompasses traditional chronic diseases that are considered of major importance because they are established as leading causes of death or morbidity [13]. The clinical domain consists of physiological conditions and factors that should be weighed when prescribing therapies (e.g., polypharmacy). The health status domain was reserved for conditions that affect function and quality of life, are likely to affect a person’s ability to adhere to therapy, and are often caused by several processes in older adults [13].

### Analytic plan

The National Center for Health Statistics (NCHS) provides sampling weights that account for sampling strategy and survey non-response. Using methods provided by NCHS, we modified the original weights in our combined sample to maintain national representation [18]. We performed analyses with statistical software designed to conduct subpopulation analyses using masked variance units to estimate appropriate standard errors. We summarize baseline characteristics using means and 95% confidence intervals. Differences in these variables between subjects with and without COPD were compared using a χ^2^ test.

We ran the analysis stratified by age group. The analyses were rerun in the subset of COPD subjects with a history of smoking to test the sensitivity of the COPD definition. To address multiple testing, we reported both the STATA-generated p-value and a Bonferroni-corrected significance level (α) [19].

All analyses were carried out in STATA version 11.1. The study protocol was approved by the Johns Hopkins University School of Medicine Institutional Review Board.

## Results

The prevalence of comorbid chronic disease among subjects with physician-diagnosed COPD was: congestive heart failure (12.1%), coronary heart disease (12.7%),hypertension (60.4%), hypercholesterolemia (47.6%), stroke (8.9%), diabetes (16.3%), osteoporosis (16.9%), arthritis (54.6%), cancer (16.5%), depression (20.6%), and anxiety (8.6%). Clinical factors potentially complicating the treatment of COPD were: dizziness or balance problems (41.1%), obesity (40.3%), urinary incontinence (34.9%), anemia (9.3%), low GFR (16.2%), use of >4 prescription medications (51.8%), and frailty (9.5%). Subjects with COPD were also found to have the following “health status” factors: memory problems (18.5%), mobility difficulty (55.6%), hearing impairment (12.1%), and visual impairment (14.0%). 96.4% of subjects with COPD had at leastone comorbidity.

Table 1 describes the basic demographic features of those with and without COPD. Those with COPD tended to be older and female.

**Table 1:** Demographics and smoking history: adults ≥45 years, with and without physician-diagnosed COPD: NHANES 1999–2008

**Demographic Variables**	**Without COPD**	**With COPD**
	**(n = 14,828)**^**a**^	**(n = 995)**^**b**^
Age, mean yr (95% CI)	60.0 (59.6–60.3)	62.7 (61.7–63.8)
**Gender**		
Male % (95% CI)	47.0 (46.2–47.9)	39.9 (36.0–44.0)
Female, % (95% CI)	53.0 (52.1–53.8)	60.1 (56.0–64.0)
**Race**		
White, % (95% CI)	76.4 (73.5–79.1)	84.6 (81.4–87.4)
Black, % (95% CI)	10.0 (8.5–11.8)	6.8 (5.1–8.9)
Hispanic, % (95% CI)	8.8 (7.0–11.0)	4.4 (3.0–6.3)
**Smoking History**		
Ever smoker, % (95% CI)	52.1 (50.7–53.4)	68.9 (65.2–72.5)
> 10 pack years, % (95% CI)	26.8 (25.7–27.9)	43.0 (38.7–47.5)

^a^ Represents ~100 million noninstitutionalized US civilians.

^b^ Represents ~10 million noninstitutionalized US civilians.

**Figure 1:**
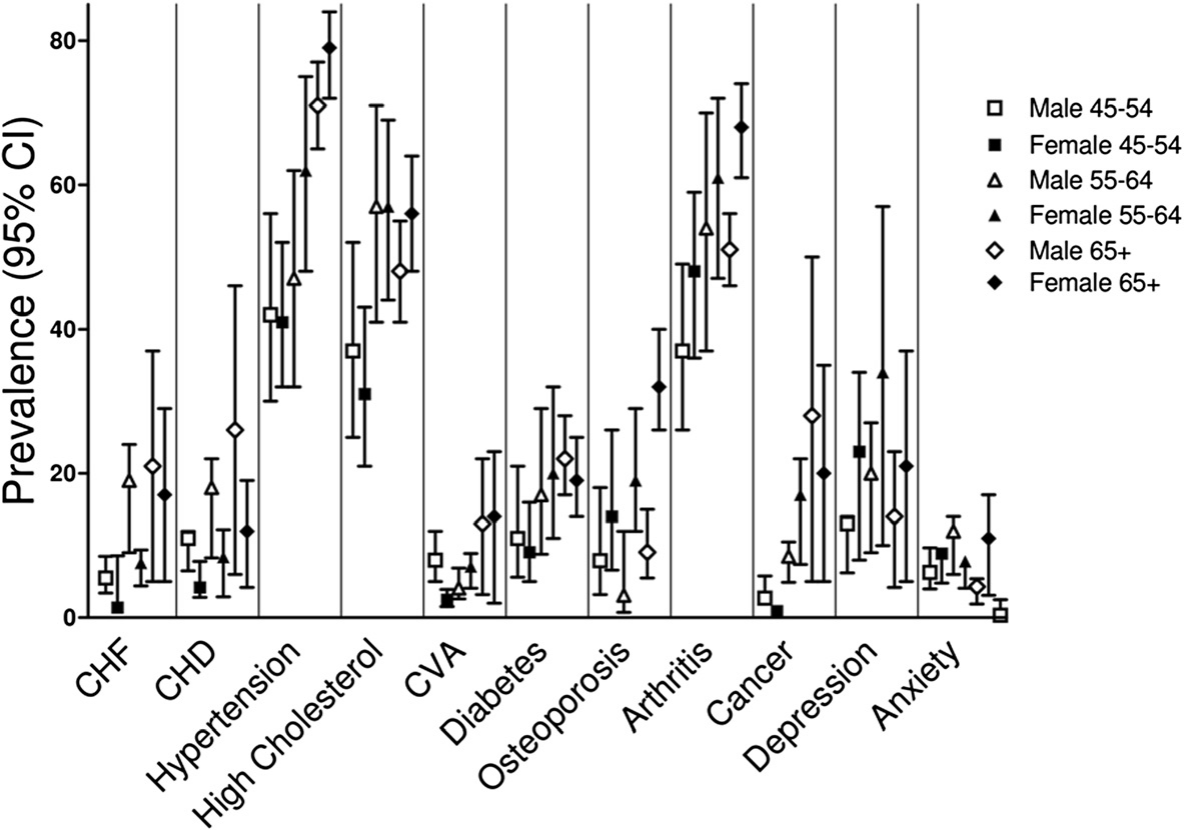
Prevalence of comorbidities stratified by age and gender among subjects with physician-diagnosed COPD: Disease Domain

**Figure 2:**
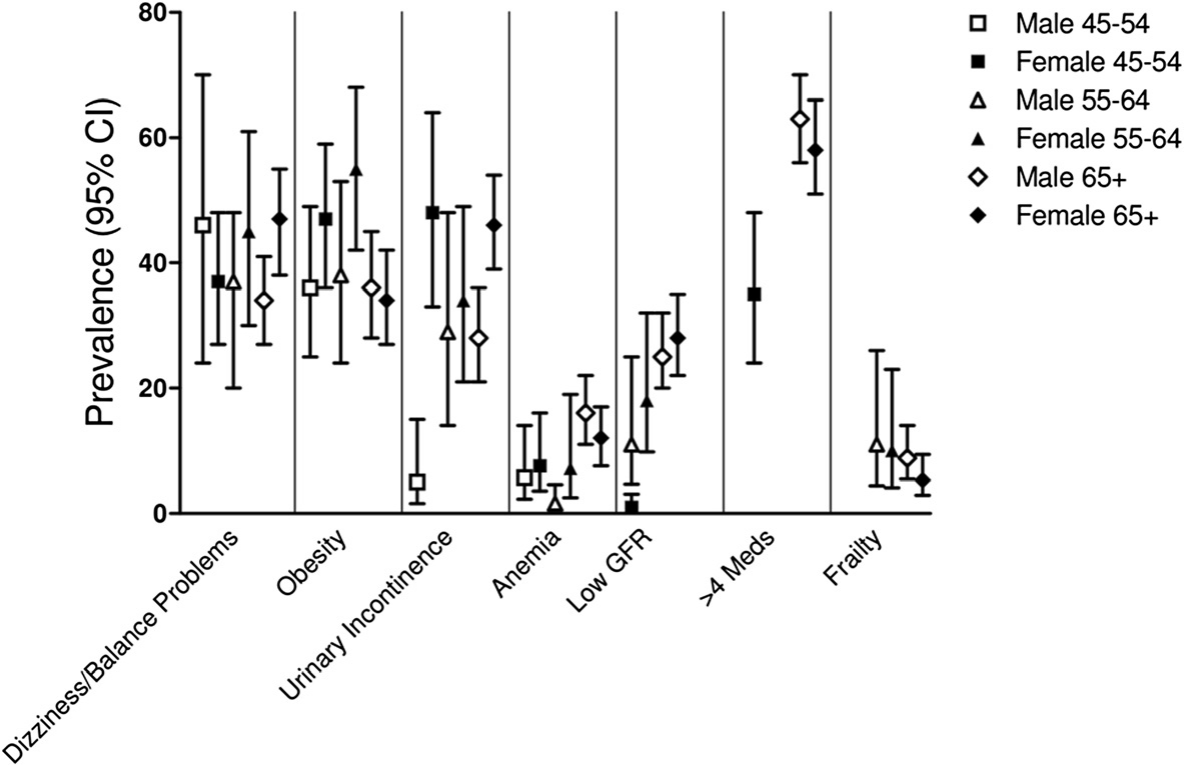
Prevalence of comorbidities stratified by age and gender among subjects with physician-diagnosed COPD: Clinical Factors

**Figure 3:**
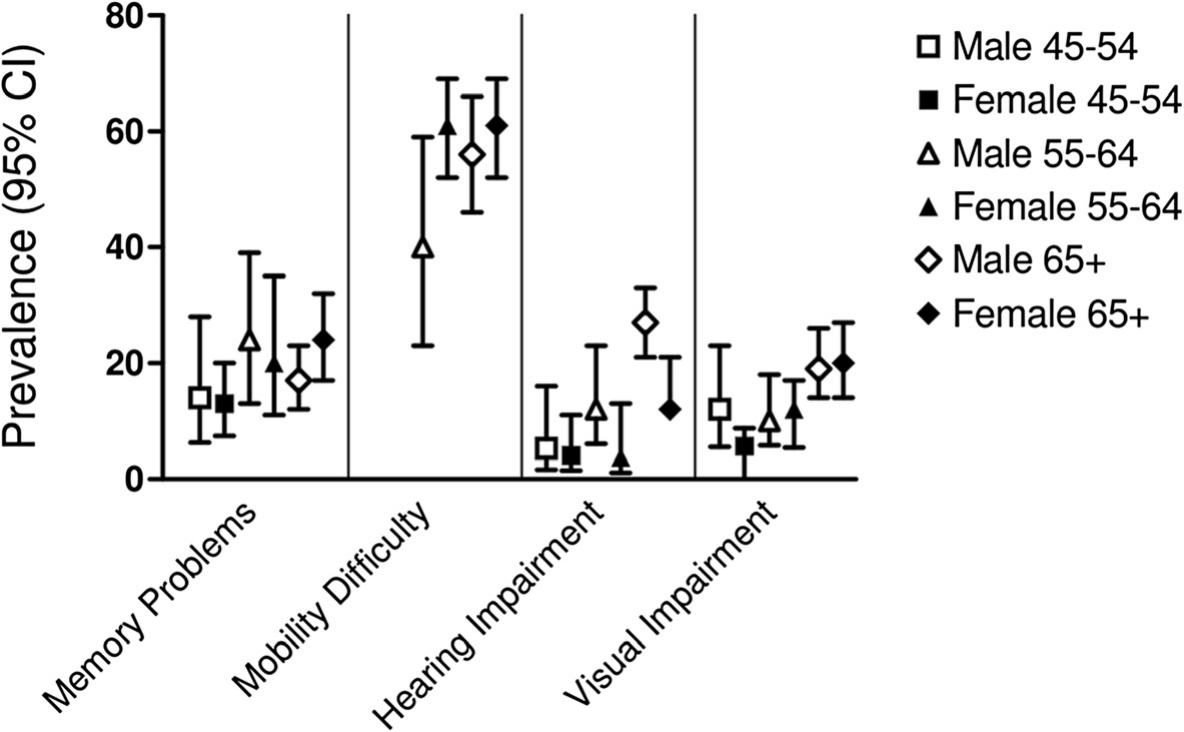
Prevalence of comorbidities stratified by age and gender among subjects with physician-diagnosed COPD: Health Status Factors

Figures 1, 2 and 3 depict the prevalence rates of conditions in the three domains (diseases, clinical factors, and health status factors) in subjects with COPD. These figures provide a visual illustration of the high prevalence of comorbidities in patients with physician-diagnosed COPD; they do not statistically compare groups. The majority of the conditions are markedly more common in the ≥65 age group than in the younger age groups. While some conditions are more common in one gender than the other (depression, CHD, osteoporosis, and hearing impairment), others, such as polypharmacy, obesity, dizziness or balance problems, and memory problems are equally common among the two genders. Missing bars on the graph represent conditions for which the sample size was small in a given gender and age group.

Table 2 compares the prevalence rates of the conditions in subjects with COPD to those without COPD. Most of the conditions are significantly more prevalent in the subjects with COPD than in the subjects without COPD. While not shown in the table, some portion of these differences can be accounted for by differences in age and gender distribution in the COPD and non-COPD groups. While not shown here, we found little difference in the prevalence of comorbid conditions between COPD subjects with and without a history of smoking,

**Table 2:** Prevalence of comorbidities: adults ≥45 with and without physician-diagnosed COPD: NHANES 1999–2008

**Conditions**	**Without COPD**	**With COPD**	**P-value**^c^
	**(n = 14,828)**^**a**^	**(n = 995)**^**b**^	
**Diseases**			
CHF, %, (95% CI)	3.9 (3.6–4.3)	12.1 (9.8–14.8)	<.0001
CHD, %, (95% CI)	6.1 (5.5–6.7)	12.7 (10.5–15.3)	<.0001
HTN, %, (95% CI)	52.2 (50.7–53.7)	60.4 (55.8–64.8)	0.0006
High cholesterol, %, (95% CI)	41.3 (39.9–42.6)	47.6 (43.0–52.2)	0.0096
CVA, %, (95% CI)	4.6 (4.2–5.0)	8.9 (7.0–11.1)	<.0001
Diabetes, %, (95% CI)	12.8 (12.1–13.7)	16.3 (13.3–19.8)	0.0184
Osteoporosis, %, (95% CI)	8.5 (7.8–9.2)	16.9 (13.7–20.7)	<.0001
Arthritis, %, (95% CI)	36.9 (35.6–38.2)	54.6 (49.8–59.3)	<.0001
Cancer, %, (95% CI)	9.9 (9.3–10.6)	16.5 (14.2–19.0)	<.0001
Depression, %, (95% CI)	12.5 (11.8–13.3)	20.6 (17.1–24.6)	<.0001
Anxiety, %, (95% CI)	3.8 (3.3–4.3)	8.6 (6.6–11.1)	<.0001
**Clinical Factors**			
Dizziness/Balance Problems, %, (95% CI)^d^	23.8 (22.4–25.4)	41.1 (36.3–46.1)	<.0001
Obesity, %, (95% CI)	33.5 (32.0–35.1)	40.3 (36.4–44.4)	0.0013
Urinary Incontinence, %, (95% CI)	27.3 (26.1–28.7)	34.9 (29.6–40.5)	0.0048
Anemia, %, (95% CI)	6.2 (5.5–7.1)	9.3 (7.1–12.0)	0.0030
Low GFR, %, (95% CI)	10.5 (9.7–11.2)	16.2 (13.3–19.5)	<.0001
>4 meds, %, (95% CI)	32.1 (30.5–33.8)	51.8 (46.2–57.2)	0.0022
Frailty, %, (95% CI)	3.1 (2.7–3.6)	9.5 (7.0–12.8)	<.0001
**Health Status Factors**			
Memory problems, %, (95% CI)	8.8 (8.1–9.5)	18.5 (14.9–22.7)	<.0001
Mobility difficulty, %, (95% CI)	32.5 (30.7–34.4)	55.6 (50.6–60.5)	<.0001
Hearing impairment, %, (95% CI)	8.3 (7.7–8.9)	12.1 (9.8–14.9)	0.0008
Visual impairment, %, (95% CI)	9.6 (9.0–10.3)	14.0 (12.0–16.2)	<.0001
**All Conditions**			
> = 1 comorbid condition, %, (95% CI)	87.7 (86.4–88.8)	96.4 (94.3–97.8)	<.0001

^a^ Represents ~100 million noninstitutionalized US civilians.

^b^ Represents ~10 million noninstitutionalized US civilians.

^c^ The Bonferroni-corrected significance level, α, is 0.0011.

^d^ Only assessed 1999–2004.

Table 3 compares the prevalence of conditions in subjects with COPD to those prevalence values found in previous publications. The table also notes the country of study, the sample size, and the sampling method. We found that, in most cases, the population-based prevalence of comorbid conditions is at least as high, if not higher, as the prevalence found in these less-generalizable COPD populations.

**Table 3:** Comparison: the prevalence of comorbidities in subjects with COPD from previous studies

**Source**	**n**	**Study location**	**Age range**	**Method of comorbidity ascertainment**	**Arthritis**	**HTN**	**Diabetes**	**Depression**	**Cancer**	**Osteoporosis**
					**(%)**	**(%)**	**(%)**	**(%)**	**(%)**	**(%)**
van Manen et al.[20]	1145	Netherlands	40+	Self-report via written survey	36	23	5	9	6	--
Mapel et al.[5]	200	New Mexico	30+	Chart abstraction	22	45	12	17 (w/other psych)	18	--
Soriano et al. [21]	2699	United Kingdom	All ages	Read codes from GPRD*	28	--	--	10 (w/other psych)	4	--
Sidney et al. [22]	45966	US, Kaiser Permanente Members	40+	ICD9 discharge codes	--	18	2	--	--	--
Walsh and Thomashow [23]	3000	US	All ages	Phone and internet surveys	70	52	16	35	4	32
Current Study	995^†^	US	45+	NHANES	54	60	16	21	16	17

*General Practice Research Database. Data collected from around 6 million primary care patients in the United Kingdom.

^†^ Nationally-representative

Adapted from Chatila et al.[9]

Reprinted with permission of the American Thoracic Society. Copyright © American Thoracic Society.

## Discussion

In this paper, we describe the prevalence of clinically-relevant comorbid conditions in a nationally-representative sample of people with physician-diagnosed COPD. We found that 96.4% of adults with physician-diagnosed COPD have at least one condition that may complicate the treatment of COPD. Most notably, 51.8% of people with COPD 45 and older are taking more than 4 medications (polypharmacy), 55.6% report mobility difficulty, 60.4% have hypertension, and 54.6% have arthritis.

These prevalence values are relatively consistent with those found in previous studies of comorbidities in COPD. However, there is a large range of previously reported prevalence values. For example, estimates of arthritis in COPD range from 22% [5] to 70% [23]. Neither of these studies, nor any other recent studies investigating comorbidity in COPD, have examined nationally-representative data. This both limits the applicability of these prevalence estimates and helps account for the large ranges in these estimates. In fact, several papers have cited lack of national representation or specific population bias as a weakness [5,8,24]. As such, our study both confirms the high prevalence of comorbidities in patients with COPD and provides specific prevalence values that are relevant on a national scale.

Another strength of our study is the range of clinically-relevant conditions assessed. While there is a lot of data on, for example, cardiovascular disease in COPD [8], there are few studies that look at the wide variety of medical conditions and functional limitations we have assessed. This is important partially because comorbidity has been found to be an important aspect of quality of life in COPD [25-27], as well as an independent risk factor for hospitalization [28]. In addition, comorbidities increase the risk of hospitalization and mortality in patients with COPD [8], and significantly increase the costs of treating COPD [29]. These conditions are also highly relevant for clinical decision-making and self-management.

The classification of the conditions into disease, clinical factor, and health status factor domains highlights that a wide range of conditions relevant to the clinical management of people with COPD are quite prevalent, and that these relevant conditions extend beyond traditionally-defined diseases.

Physicians must be judicious when caring for patients with COPD. The high prevalence of comorbidity and polypharmacy means physicians must be cognizant of potential adverse drug events and nonadherence. People with COPD are often complex, and, thus, we will need to improve our ability to prioritize treatment recommendations based on relative benefits and harms and patient preferences. Current guidelines, and our evidence base, do not yet adequately inform this critical clinical decision-making [13].

Clinical practice guidelines generally do not address how to treat COPD in the context of comorbid conditions [11]. As such, these guidelines may be of little help when dealing with the majority of COPD patients [30,31]. For example, β-blockers, which are indicated for cardiovascular disease, may worsen lung function in some patients with COPD; some studies, however, have shown that this is not a contraindication to the initiation of β-blockers [32]. Conversely, bronchodilators, which are believed to be beneficial for pulmonary function, may worsen tachyarrhythmias [9]. Guidance about these potential interactions, and the quality of evidence supporting any recommendations about them, would be very useful to clinicians. Our results can inform clinical practice guideline priority-setting processes to determine which comorbidities should be addressed in future COPD guidelines.

It is imperative that therapeutic trials be designed to reflect the *true* population of people with COPD. Many studies exclude patients with significant comorbid conditions [33]. Herland et al. found that only 17% of a group of COPD patients would be eligible for a “typical” clinical trial. 65.9% of COPD patients in this study were “excluded” due to significant comorbidities, including diabetes, depression, and ischemic heart disease [34]. These exclusions may be troublesome given the high prevalence of these diseases in people with COPD, and the potential for interactions between the diseases and their treatments. Our study highlights the need for future clinical trials to evaluate safety and effectiveness in COPD patients with multiple comorbidities.

There is evidence that treating COPD may benefit the course of comorbid conditions, and, visa-versa [35]. For example, several observational studies have shown improved outcomes in COPD patients treated with statins [36,37], independent of whether patients have a comorbid diagnosis of ischemic heart disease [38]. While a randomized controlled trial has shown an improvement in exercise tolerance in COPD patients treated with statins [39], more prospective intervention trials are needed to look at the use of statins in COPD [40].

Given these potential complications of treatment and the interactions between comorbid conditions and COPD, some researchers have started to advocate for an integrated-care approach to the management of patients with COPD. Sonetti et al. in a recent review advocate for a chronic care model approach to COPD management, with an approach that includes automatic screening for common comorbidities [41,42]. In order to truly move to such a system, however, it is necessary to have a good understanding of how best to treat COPD in the context of comorbid conditions. First steps to accomplishing this are (a) determining common “patterns” of co-existing conditions (b) including patients with comorbid conditions in clinical trials with appropriate analytic strategies to understand heterogeneity of treatment effect [43] (c) evaluating current treatment regimens in patients with different patterns of comorbid conditions and (d) continuing to study possible pathophysiologic connections between COPD and comorbidities. Further research should also continue to explore the effects comorbid conditions have on outcomes (health-related and other) in COPD.

### Limitations

As spirometry data is not available in NHANES 1999–2006, we were not able to look at comorbidities in the context of the severity of COPD. This is significant because a recent study showed that increased respiratory impairment was associated with a higher risk of having comorbid hypertension, CVD, and diabetes [8].

Also due to the absence of spirometry data, we defined COPD via self-report. While this does not meet the gold standard definition for COPD (which is spirometric) [5], we are aware of no other applicable datasets that are nationally-representative and as comprehensive and current as NHANES 1999–2008. For example, NHANES 1988–1994 has spirometry data on a subset of participants; but, given the changing demographics of the US, it is unlikely this data is entirely representative of the current US population.

We acknowledge that the prevalence of comorbidities in physician-diagnosed COPD may be different than that in spirometrically-defined COPD; for example, patients with a higher burden of disease and lower health status may be more likely to receive a physician diagnosis of COPD. There is also likely misclassification of some subjects - subjects with spirometrically-defined COPD in the group without physician-diagnosed COPD, and subjects who reported a physician-diagnosis of COPD, but who would not meet spirometric criteria. Given the available data, and the desire to assess comorbidities in a larger sample of adults across multiple waves of NHANES, we believe physician-diagnosed COPD is a relevant outcome.

A study by Barr et al. found that self-report-based surveys are an appropriate way to study respiratory disease in healthcare professionals [44], and many studies of comorbidities in COPD have used self-report [20]. We found little difference in the prevalence of comorbid conditions between COPD subjects with and without a history of smoking, which helps validate our definition of COPD. Our definitions of many of the comorbidities were similarly limited by the need to, in some cases, define conditions by self-report.

We were also limited by factors included in the NHANES data set. We would have liked to assess warfarin use in COPD patients (our sample size was too small), human immunodeficiency virus (NHANES only runs HIV tests on subjects between the ages of 18 and 49), insomnia/sleeping problems [27] (only assessed in NHANES 2005–2008), gastroesophageal reflux disease, pulmonary embolism, and pneumonia (these conditions were not directly assessed in NHANES).

## Conclusions

Comorbid conditions are the rule, not the exception, in patients with physician-diagnosed COPD. While 96.4% of adults 45 and older with COPD have at least one condition that may complicate the treatment of COPD, few trials or practice guidelines take these conditions into consideration. Describing the nationally-representative prevalence of comorbid conditions in patients with physician-diagnosed COPD is the first step towards developing an evidence base, and clinical practice guidelines, that better represent the true population of patients with COPD.

## Competing interests

Dr. Bruce Leff has served on a strategic advisory board to Amedisys Inc and has consulted with Intersection LLC via a consulting agreement between Intersection LLC and Johns Hopkins Medicine. All other authors declare that they have no competing interests.

## Authors’ contributions

KS carried out the initial literature search, contributed to conception and design, completed the data analysis and interpretation of data, and drafted and edited the manuscript. COW provided many of the condition definitions, contributed to the statistical analysis and interpretation of data, and revised the manuscript critically for important intellectual content. TL and JAK provided initial conceptual guidance, contributed to the study design, and revised it critically for important intellectual content. BL and JLW helped with initial conceptual guidance, aided in condition definitions, and revised it critically for important intellectual content. CB helped guide the initial literature search, contributed to the conception and design, helped with the data analysis and interpretation of data, and contributed significantly to the drafting, organization, and conclusions of the manuscript. All authors read and approved the final manuscript.

## Pre-publication history

The pre-publication history for this paper can be accessed here:

http://www.biomedcentral.com/1471-2466/12/26/prepub
